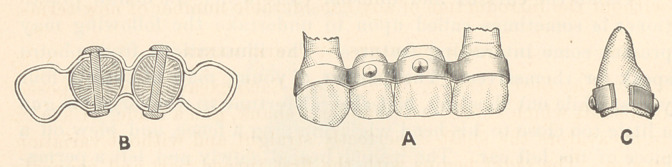# Etruscan Bridge-Work

**Published:** 1898-05

**Authors:** Henry H. Burchard


### ETRUSCAN BRIDGE-WORK.

BY HENRY H. BURCHARD, M.D., D.D.S.

FIGS. A, B, C represent a specimen of Etruscan dentistry, the prototype of a modern variety of bridge-work. It consists of a continuous band of gold crimped into the interspaces between the teeth, to secure close adaptation. Two natural teeth are cut out, curved beneath, to fit them to the natural gums, as shown in C.

B

A

C

The teeth are secured to the band by means of two button-head rivets piercing both band and teeth, as shown in B. In A, the piece is represented in position for which it is intended. The color of the gold in the specimen indicates an alloy of gold and silver, 22 to 23 carats fine.

The drawing was made from specimens, Nos. 10,334 and 10,335, of the Mayer collection of antiquities in the Brown Museum, Liverpool, England. They are probably the same referred to by Dr. W. H. Waite.—*Independent Practitioner*, vol. vi., April, 1885, p. 195.

## Figures and Tables

**Figure f1:**